# Duration of Untreated Disorder and Cannabis Use: An Observational Study on a Cohort of Young Italian Patients Experiencing Psychotic Experiences and Dissociative Symptoms

**DOI:** 10.3390/ijerph182312632

**Published:** 2021-11-30

**Authors:** Valerio Ricci, Giovanni Martinotti, Franca Ceci, Stefania Chiappini, Francesco Di Carlo, Julius Burkauskas, Ottavia Susini, Debora Luciani, Diego Quattrone, Domenico De Berardis, Mauro Pettorruso, Giuseppe Maina, Massimo Di Giannantonio

**Affiliations:** 1 Department of Neuroscience, San Luigi Gonzaga University Hospital, 10043 Orbassano, Italy; v.ricci@sanluigi.piemonte.it (V.R.); giuseppe.maina@unito.it (G.M.); 2 Department of Neurosciences, Imaging and Clinical Sciences, Università degli Studi G. D’Annunzio Chieti-Pescara, 66100 Chieti, Italy; giovanni.martinotti@gmail.com (G.M.); franca.ceci@live.it (F.C.); francesco.dic@hotmail.it (F.D.C.); susiniottavia@gmail.com (O.S.); debora.luciani@studenti.unich.it (D.L.); mauro.pettorruso@hotmail.it (M.P.); digiannantonio@unich.it (M.D.G.); 3 Psychopharmacology, Drug Misuse and Novel Psychoactive Substances Research Unit, School of Life and Medical Sciences, University of Hertfordshire, Hatfield AL10 9AB, UK; 4 Laboratory of Behavioral Medicine, Neuroscience Institute, Lithuanian University of Health Sciences, 00135 Palanga, Lithuania; julius.burkauskas@lsmuni.lt; 5 Social, Genetic and Developmental Psychiatry Centre, Institute of Psychiatry, Psychology and Neuroscience, King’s College London, London SE5 8AF, UK; diegoquattrone@me.com; 6 NHS, Department of Mental Health, Psychiatric Service for Diagnosis and Treatment, Hospital “G. Mazzini”, ASL 4, 64100 Teramo, Italy; dodebera@aliceposta.it

**Keywords:** DUP, cannabis, dissociation, first-episode psychosis, schizophrenia

## Abstract

Background: The Duration of Untreated Psychosis (DUP) is the time between the first-episode psychosis (FEP) and the initiation of antipsychotic treatment. It is an important predictor of several disease-related outcomes in psychotic disorders. The aim of this manuscript is investigating the influence of cannabis on the DUP and its clinical correlates. Methods: During years 2014–2019, sixty-two FEP patients with and without cannabis use disorder (CUD) were recruited from several Italian psychiatric hospitals. The subjects were then divided into two groups based on the duration of the DUP and assessed at the beginning of the antipsychotic treatment and after 3 and 6 months, using the Positive and Negative Syndrome Scale (PANSS), the Global Assessment of Functioning (GAF) scale, and the Dissociative Experiences Scale (DES-II). Results: As expected, a longer DUP was associated with worse symptoms and cannabis use did not seem to affect the DUP, but both were related with more dissociative symptoms at onset and over time. Discussion: According to our study, cannabis use can be a predictor of FEP and DUP, and of disease outcome. However, several factors might influence the relationship between cannabis use and DUP. Preventing cannabis use and early diagnosis of psychotic disorders might impact the disease by reducing the persistence of symptoms and limiting dissociative experiences.

## 1. Introduction

### 1.1. Psychosis and Duration of Untreated Psychosis (DUP)

The term psychosis defines a functionally disruptive symptom of many psychiatric, neurodevelopmental, and neurological conditions, described as a significantly altered or distorted perception of reality, together with hallucinations (false perceptions), delusions (false beliefs), and/or disrupted or disorganized thinking [1]. According to the fifth edition of the Diagnostic and Statistical Manual of Mental Disorders (DSM-5), *psychotic disorders* are characterized by delusions, hallucinations, disorganized thinking, disorganized motor behaviour, and negative symptoms [2]. Current treatments and clinical interventions are effective in reducing these symptoms; however, psychotic disorders still rank among the major causes of disability [3]. 

The early stage of the disease is considered a critical period in determining its long-term outcome [4] in relation to personal, familiar, social, and clinical burdens. While the first-episode psychosis (FEP) refers to the first time someone experiences a psychotic episode, the duration of untreated psychosis (DUP) is the time that elapses between the first experience of psychosis and the initiation of antipsychotic treatment [5]. Current literature proposes a distinction between long and short DUP; however, a time cut-off has not been established for making such a distinction. Depending on the study, the time cut-off ranges from 3 [6] to 18 months [7], and includes 6 months [8], 9 months [5] and 12 months [9]. Several factors might contribute to longer DUP, such as: (i) low awareness of psychotic symptoms; (ii) dismissive attitude of patients and parents experiencing prominent negative symptoms and social withdrawal as opposed to positive symptoms; and (iii) health care providers’ failure to recognize psychosis [10]. A longer DUP is associated with negative clinical outcomes [11], which may include a presentation with more severe positive [12] and negative symptoms [13,14,15], worse social functioning [12,16], and lower response to the pharmacological treatments with specific antipsychotics [11,17]. Dissociative symptoms might be considered part of the psychotic process itself [18]; in fact, conversely, certain psychotic symptoms might be better conceptualized as dissociative in nature [19], and, in both cases, their origin could be related to a traumatic experience. A robust and well-replicated relationship exists between dissociative experiences and all symptoms of psychosis, specifically positive symptoms, e.g., hallucinations [19], paranoia, and delusions, which are consistent with models of traumatic memory and associated with powerful feelings of depersonalization/derealization, subsequently driving the development of delusions and other psychotic symptoms. With regard to this, the DSM-5 defines the dissociation as the “disruption and/or discontinuity in the normal integration of consciousness, memory, identity, emotion, perception, body representation, motor control and behaviour”. Several psychiatric disorders can manifest with dissociative symptoms that are usually linked with poor response to the drug treatment and, therefore, with a worse outcome [20,21]. Despite this, to date the relationship between longer DUP and dissociation symptoms in psychiatric disorders has been scarcely examined.

### 1.2. Cannabis Use and First Episode Psychosis (FEP)

Cannabis is the most widely consumed drug worldwide, despite being illegal in many countries [19]. In fact, it has been estimated that in 2020 the 27.2% of adults in the European Union aged 15–64 have used cannabis at least once in their lifetime, and that the 15.4% of young adults aged 15–34 have used cannabis in the last year [22]. Heavy use of cannabis might have a negative impact on mental health and functioning [23], and it may result in a condition known as cannabis use disorder (CUD) [24]. In the long term, a CUD can result in several medical and psychiatric morbidity, poor cognitive performances, dysfunctional social behaviour, and legal consequences [25]. Frequent use of cannabis is also associated with an increased risk of developing psychotic symptoms [26,27,28,29,30], their course exacerbation [26,31], and finally, an enhanced possibility of a psychotic relapse [32]. It was found to be the strongest predictor of relapse over one year compared with other risk factors including adherence to drug therapy, DUP, chronic and acute stress, and emotional expression [32]. Products that are increasingly widespread on the market, such as high-potency cannabis (HPC) and synthetic cannabinoids, are correlated with a high risk of developing psychotic and dissociative symptoms, as well as worse long-term outcomes [33,34,35]. 

Currently, the scientific literature disagrees on whether cannabis-users or non-users have a different duration of DUP: in fact, some studies suggested that FEP may occur earlier in cannabis users than in non-users [36,37], while others recorded that cannabis use is associated with a longer DUP [38]; and, finally, a meta-analysis found no differences in DUP between cannabis users and non-users [39].

Aim of the study: The main objective of the study was to examine whether cannabis use affects the duration of the DUP on a sample of young adults having an FEP, both with and without CUD. Secondly, as in many of the previous studies on this topic, after differentiating patients according to the duration of the DUP (less or more than one year), we decided to assess whether the duration of the DUP affected the appearance of both psychotic and dissociative symptoms and the overall functioning at the initiation of the antipsychotic treatment and three and six months after it. The same parameters were evaluated at the same times chosen within the two groups of patients with short and long DUP, distinguishing them based on whether or not they were affected by CUD. Finally, we verified if it was a correlation between the scores of the Positive and Negative Syndrome Scale (PANSS), Global Assessment of Functioning (GAF) and Dissociative Experiences Scale-II (DES-II) scales in the three evaluation periods and the duration of the DUP in weeks in the whole sample.

## 2. Materials and Methods

### 2.1. Participants

All subjects included in this study were recruited in psychiatric hospitals in Piemonte (Italy) in the period between January 2014 and December 2019, with an average of 15 patients per year. All had experienced an FEP, defined as the first time a patient showed positive psychotic symptoms of delusion and/or hallucinations or marked disorganized behaviour. During the follow up, 4–5 patients per year were lost with an attrition rate of approximately 33%.

The inclusion criteria were (1) age between 16 and 40 years; (2) diagnosis of schizophrenia spectrum or other psychotic disorders (performed at baseline and confirmed at six months), with or without concurrent CUD, according to the diagnostic criteria of the DSM-5; (3) no previous use of cannabis for individuals without CUD; and (4) residence in the area of recruitment around Turin. The exclusion criteria were (1) previous contact with mental health services for psychosis; (2) prior treatment with antipsychotic medication; (3) a diagnosis of intellectual disability (intelligence quotient below 70); (4) any lifetime history of significant medical illnesses; (5) non-sporadic use (greater than once or twice a month) of substances other than cannabis (cocaine, heroin, 3,4-methylenedioxymethamphetamine [MDMA], and ketamine); (6) alcohol use disorder. After receiving a complete description of the study, approximately 62 participants gave informed written consent and were consecutively recruited over a period of 3 years. 

All patients received antipsychotic treatment in line with clinical guidelines. They were evaluated at the beginning of drug treatment (T0) and prospectively 3 (T1) and 6 months (T2) after it. The use of cannabis was detected through clinical interviews to patients and their families. The DUP was measured (in weeks) as the time elapsed from the onset of key symptoms (hallucinations, delusions, or bizarre behaviour) to the beginning of treatment (pharmacotherapy/psychotherapy) prescribed by a psychiatrist. This evaluation was made with the Italian version of the Early Recognition Inventory Retrospective Assessment of Symptoms checklist (ERIraos-CL) [40], a 17-item screening checklist intended to select persons needing a more in-depth assessment. The ERIraos-CL has 17 items designed to assist the exploration of individual proneness to schizophrenia, including items about changes in perception and thought interference and 2 items about paranoid ideation and hallucinations, which are more clearly indicative of psychosis [41,42].

The sample was divided in 2 groups based on the duration of the DUP using an arbitrarily set cut-off of 1 year:

- a group with a DUP < 1 year, considered as short DUP;
- a group with a DUP ≥ 1 year, considered as long DUP.

Within each group, patients were further subdivided based on whether they had CUD:

- a group comprising patients with persistent use of cannabis in quantities equal to or greater than 15 joints per week who met the DSM-5 diagnostic criteria for CUD (CUD+ group);
- a group comprising patients without a prior history of CUD (CUD− group).

The study was approved by the SS. Annunziata Hospital—University G. D’Annunzio Ethical Committee (P.N.189, 26 January 2012).

### 2.2. Measurements

The following psychopathological scales were administered to the patients at T0, T1 and T2:

- PANSS [43] is based on two established psychiatric rating systems and is a 30-item questionnaire conceived as an operationalized, drug-sensitive instrument that provides balanced representation of positive and negative symptoms and gauges their relationship to one another and to global psychopathology. It thus constitutes 3 subscales (i.e., positive, negative, and psychopathology) measuring positive and negative syndromes and general severity of illness [43];
- The GAF scale [44] is a single measure of overall psychosocial impairment caused by mental factors, constituting Axis V of the DSM, third and fourth versions. It is a clinician-rated scale for evaluating the level of psychological, social, and occupational functioning on a continuum from 0 to 100 [45];
- The DES-II [46] is a self-report questionnaire that measures dissociative experiences, such as derealization, depersonalization, absorption, and amnesia. The DES-II has been prevalently used as a screening tool in patients suffering from psychotic disorders or schizophrenia to evaluate the dissociative experience [47]. The DES [48] comprises 28 items based on the assumption of a ‘dissociative continuum’ ranging from a mild alteration to severe dissociation. Subjects are asked to select their choices for topics, such as experiences of amnesia, absorption, depersonalization, and derealization. Cronbach’s alpha for this instrument in the present sample was 0.812, suggesting a good internal consistency.

### 2.3. Statistical Analysis

All statistical analyses were performed using IBM SPSS windows version 22. The Shapiro–Wilk test and estimation of the values of asymmetry and excess coefficients were used to determine whether the data were normally distributed in the sample of the whole group and in the sub-groups (based on DUP duration and Cannabis use criteria). Groups of patients with DUP < 1 year and ≥ 1 years were compared using Student’s *t*-test, Chi-square test and Fisher’s exact test, as appropriate. Student’s *t*-test was used for continuous variables, whereas Chi-square test and Fisher’s exact test were used for categorical variables. Pearson’s correlation coefficient was used for analyzing the relationships between continuous variables in the whole sample. The quantitative parameters were presented as mean ± standard deviation (SD) and the qualitative parameters as number and percentage per class. The significance level has been set for *p* < 0.05.

## 3. Results

### 3.1. Demographical and Clinical Characteristics of the Sample

A total of 62 patients completed all of the follow-up assessments and were included in the analysis, with a mean age of 22.9 years (SD ± 3.9). Of these, half of the sample (*n* = 31) had a DUP < 1 year, and the remanent part (*n* = 31) had a DUP ≥ 1 year. Mean DUP in the former group was 27.4 weeks (SD ± 15.8), while in the latter was 133.7 weeks (SD ± 133.7). The two groups were comparable in terms of age (*p* = 0.392) and gender (*p* = 0.611). Within each group, approximately half of the patients had a current CUD, without differences between the groups (*p* = 1.00). Most of the participants were inpatients (*n* = 52, 83.9%). The participants’ characteristics at baseline are detailed in Table 1.

### 3.2. Cannabis Use and Duration of Untreated Psychosis

DUP weeks were compared among patients with and without CUD. No significant differences were found between the groups (*p* = 0.909) (see Table 2).

### 3.3. Psychometric Scores

Psychometric scores at baseline (T0), T1 and T2 were compared between the two groups. PANSS positive and negative subscales were higher among patients with DUP ≥ 1 year both at baseline and at follow-ups. GAF scored higher among patients with higher DUP only at T2; DES scores were significantly higher among DUP ≥ 1 year group from T0 to T2. The psychometric results are reported in Table 3. 

Correlations between weeks of DUP and psychometric score were studies using Pearson’s correlation coefficient. The strongest correlations were found between weeks of DUP and PANSS negative scores. Pearson’s correlation results are shown in Table 4.

### 3.4. Psychometric Scores and Cannabis Use

Patients were then split into two groups on the basis of their current CUD. Among patients with DUP < 1 year, CUD+ patients scored higher at PANSS positive subscale and DES at baseline, T1 and T2. GAF scored significantly higher among CUD− patients at T0 and T1. Regarding patients with DUP ≥ 1 year, negative symptoms as measured by PANSS were higher among CUD− patients at T0, T1 and T2. GAF at T2 scored higher among CUD− patients, while DES scores were higher among those with a CUD. Results are detailed in Table 5.

## 4. Discussion

A merit of this study is that it increases knowledge of a topic on which there is conflicting evidence and also provides an opportunity for further investigation in the future. Indeed, it investigates the influence of cannabis use on the DUP on a sample of young adults with an FEP. In addition, we assessed how DUP duration may affect the extent of the psychotic and dissociative symptoms, as well as the level of general functioning of patients, during an initial assessment and in different follow-ups. Finally, the results obtained in relation to dissociative symptoms constitute an important novelty. 

The main result that we observed is the absence of any significant differences in the duration of DUP between CUD+ patients and CUD− patients. This neutral result regarding cannabis use is consistent with the literature, where data still appear to be heterogeneous, without a clear direction able to differentiate cannabis users versus cannabis-free patients [38,39]. However, we have hypothesized that cannabis has a complex and heterogeneous effect on psychosis clinical presentation and is probably able to determine both a reduction and an increase of the DUP so as to determine a neutral effect. In fact, on the one hand, DUP could be shortened by acute positive psychotic symptoms in cannabis users who may arrive earlier to clinical observation in emergency settings. This is consistent with studies showing that cannabis use may result in a rapid onset of psychotic symptoms, requiring a prompt intervention [35,37]. On the other hand, the use of cannabis could have made DUP longer for different reasons [26], including: (i) self-medication before the development of a thriving and full-blown symptomatology [34,48]; (ii) patients’ delay in seeking help due to the perception of cannabis as a safe substance or unwillingness to disclose cannabis addiction [49,50,51]; and (iii) clinicians’ failure to recognize psychotic symptoms which may be confounded by cannabis intoxication [52,53,54].

Furthermore, it is important to acknowledge that different types of cannabis may differently impact on DUP. The use of low potency cannabis might perhaps constitute self-treatment and extend the DUP, whereas the use of HPC or synthetic cannabinoids result in more prominent [33,34] psychotic symptoms that often require emergency treatment. Finally, a further explanation could rely on a clinical assessment made by an addiction specialist or a general practitioner not adequately trained in the evaluation of psychotic episodes, who may have assessed the patient underestimating the psychopathological problem. This may by a typical situation in Italy, where addiction specialists are not required to have a psychiatric background.

Moreover, we found that a longer DUP was associated with more severe positive and negative psychotic symptoms at the beginning of pharmacological treatment and three and six months after it. These findings are consistent with a large number of studies [12,14]. The total psychopathology score at the PANSS was also higher in patients presenting with a longer DUP. In all cases, however, there was an improvement in symptoms over time, which is also in line with previous literature [26]. The strong correlation between the DUP in weeks and a greater severity of negative symptoms has already emerged from other studies [13,14]. This result could lead to hypothesize the development of structural and functional brain [8,55] alterations responsible for a greater persistence of negative psychotic symptoms during longer periods of untreated psychosis.

The overall functioning of patients measured with the GAF scale also improved over time in both patient groups, but it was always worse over the three time points examined in patients with the longest DUP. This result is also in line with previous studies [12,16].

Of particular interest in our study are the findings on dissociative symptoms, which we report for the first time to be associated with a longer DUP. This might possibly be related to the fact that both positive and negative psychotic symptoms and overall functioning are affected by the DUP, but also that the dissociative dimension of psychosis is worsened at onset and over time by a longer DUP.

Finally, by evaluating the influence of cannabis consumption in the two groups of patients with DUP < 1 year and DUP ≥ 1, it can be observed that in the first case the CUD + patients had significantly greater positive and dissociative symptoms at the three time points examined, as well as worse functioning at the start of treatment and after three months. Conversely, in the second group of patients there were the following results: (i) a significant difference in negative symptoms at the first evaluation and over time, which would be greater in CUD− patients; (ii) a worse functioning at six months in CUD+ patients; and (iii) a greater dissociative symptomatology in the three evaluations in CUD+ patients. With regard to these, we may highlight two points:

- the greater negative symptomatology represented by patients with DUP ≥ 1 year and CUD−, which could be explained by a greater intensity and persistence of positive symptoms in CUD+ patients, therefore masking the negative ones, being a longer DUP determining the intensity and persistence of negative symptoms [56];
- the major dissociative symptomatology presented by CUD+ patients both in the case of short and long DUP. The presence of a high level of dissociative symptoms in FEP and in general in psychosis is consistent with the current literature and recently investigated by our group, that showed how dissociation is a typical feature of schizophrenia spectrum disorder associated with CUD [19,57]. This form of psychosis associated with substance use represents a modern clinical presentation of schizophrenia spectrum disorder, also frequently observed in adolescents and young adults, as recently reported [19,58].

### Limitations

Different limitations may have affected the interpretation of our study data: (1) the study included a low number of participants for a disorder that is heterogenous in its clinical symptoms; (2) a period of six months cannot be considered sufficient to draw conclusions with regard to clinical outcomes; (3) the pharmacological treatment included a wide variety of antipsychotic treatments; (4) cannabis use was obtained through clinical interviews with patients and family members; (5) the type of cannabis used has not been investigated; and (6) finally, regarding the dissociative experience, the DES questionnaire used refers to a self-report measure, and so, to a subjective experience which might have been under- or over-estimated, or possibly, misunderstood and related to a psychotic symptomatology.

## 5. Conclusions

Despite limitations, the present study shows that cannabis use may be a predictor of FEP and DUP, and of disease outcome in psychotic disorders. However, several factors might influence the relationship between cannabis use and DUP and several factors remained unclear. Further studies are needed that investigate the use of cannabis in a more structured way in terms of frequency, quantity, and type. Moreover, longer observation periods and integration of clinical data with structural neuroimaging and Functional Magnetic Resonance Imaging (fMRI) data would be important to evaluate possible biological correlates. Finally, early diagnosis and interventions, including prevention of cannabis use, are needed to improve patient outcome in psychotic disorders [59]. 

## Figures and Tables

**Table 1:** Demographic and clinical characteristics in the sample (*n* = 62).

Variables	DUP < 1 Year (N = 31)	DUP ≥ 1 Year (N = 31)	Statistics	*p*
**Age in years (SD)**	21.6 (3.5)	22.4 (4.4)	−0.862	0.392 ^a^
**Gender, female, n (%)**	14 (45.2)	16 (51.6)	0.58	0.611 ^b^
**Current cannabis use disorder, n (%)**	16 (51.6)	16 (51.6)	0.000	1.000 ^b^
**Marital status, n (%)**			2.200	0.333 ^b^
Not married	24 (77.4)	21 (67.7)		
Married	6 (19.4)	10 (32.3)		
Divorced	1 (3.2)	0 (0)		
**Educational level, n (%)**			0.404	0.817 ^b^
Student	11 (35.5)	11 (35.5)		
Worker	10 (32.3)	12 (38.7)		
Unemployed	10 (32.3)	8 (25.8)		
**Care setting, inpatients, n (%)**	26 (83.9)	26 (83.9)	0.000	1.000 ^b^
**Suicide attempts, n (%)**	5 (16.1)	4 (12.9)		1.000 ^c^
**Other substances sporadic use, n (%)**	20 (64.5)	16 (51.6)	1.060	0.303 ^b^
**Smoking, n (%)**	16 (51.6)	20 (64.5)	1.060	0.303 ^b^

Statistics: ^a^ Student’s *t*-test, ^b^ chi-square test, ^c^ Fisher Exact two-tailed test.

**Table 2:** Duration of untreated psychosis and cannabis use.

Variables	CUD+ (N = 32)	CUD− (N = 30)	Statistics	*p*
DUP in weeks (SD)	77.4 (77)	81.7 (77.7)	0.115	0.909

Statistics: Student’s *t* test.

**Table 3:** Psychometric scores (PANSS, GAF, DES) at different times in the two groups.

Total Patients N = 62	DUP < 1 Year (N = 31)	DUP ≥ 1 Year (N = 31)	T	*p*	Effect Size (D)
**PANSS—positive**					
T0	27.45 ± 4.01	31.16 ± 5.39	−3.075	0.003	0.78
T1	25.10 ± 4.55	29.10 ± 4.92	−3.323	0.002	0.84
T2	23.03 ± 4.76	28.16 ± 5.42	−3.959	<0.001	1.01
**PANSS—negative**					
T0	21.52 ± 3.41	26.74 ± 4.43	−5.204	<0.001	1.32
T1	20.13 ± 2.99	25.03 ± 4.29	−5.220	<0.001	1.33
T2	18.97 ± 2.73	24.52 ± 4.07	−6.311	<0.001	1.60
**PANSS—general**					
T0	59.19 ± 9.75	58.10 ± 5.90	0.536	0.594	0.14
T1	54.16 ± 9.67	55.68 ± 6.92	−0.710	0.481	0.18
T2	50.23 ± 10.14	53.77 ± 6.33	−1.653	0.104	0.42
**PANSS—total**					
T0	108.16 ± 11.51	116.00 ± 8.36	−3.069	0.003	0.78
T1	99.39 ± 11.21	109.81 ± 9.74	−3.908	<0.001	0.99
T2	92.23 ± 11.78	106.45 ± 8.94	−5.354	<0.001	1.36
**GAF**					
T0	48.61 ± 4.88	48.32 ± 5.87	0.212	0.833	0.05
T1	52.68 ± 4.32	50.45 ± 5.31	1.810	0.075	0.46
T2	58.61 ± 4.11	54.68 ± 3.28	4.165	<0.001	1.06
**DES**					
T0	29.65 ± 6.05	34.29 ± 7.56	−2.671	0.010	0.68
T1	26.19 ± 6.63	33.06 ± 7.82	−3.733	<0.001	0.95
T2	25.16 ± 6.77	30.16 ± 8.60	−2.544	0.014	0.65

Statistics: Student’s *t*-test; all results are reported as mean ± SD.

**Table 4:** Pearson’s correlation between DUP and psychometric scales.

	DUP in Weeks
**PANSS—positive**	
T0	0.137 (0.287)
T1	0.152 (0.239)
T2	0.218 (0.089)
**PANSS—negative**	
T0	0.381 (0.002)
T1	0.375 (0.003)
T2	0.469 (<0.001)
**PANSS—general**	
T0	−0.041 (0.749)
T1	0.059 (0.649)
T2	0.103 (0.427)
**PANSS—total**	
T0	0.202 (0.116)
T1	0.251 (0.049)
T2	0.333 (0.008)
**GAF**	
T0	0.013 (0.921)
T1	−0.146 (0.258)
T2	−0.284 (0.025)
**DES**	
T0	0.157 (0.224)
T1	0.221 (0.084)
T2	0.035 (0.788)

All results are reported as r coefficient (*p*).

**Table 5:** Psychometric scores (PANSS, GAF, DES) at different times in the two groups, divided for CUD.

**DUP < 1 year**		**CUD− (*n* = 15)**	**CUD+ (*n* = 16)**	***t***	***p***
	**PANSS—positive**				
	T0	26.00 ± 2.36	28.81 ± 4.78	−2.097	0.048
	T1	22.93 ± 3.75	27.13 ± 4.38	−2.853	0.008
	T2	20.13 ± 3.74	25.75 ± 3.99	−4.036	0.000
	**PANSS—negative**				
	T0	20.40 ± 3.09	22.56 ± 3.44	−1.836	0.077
	T1	19.20 ± 2.37	21.00 ± 3.31	−1.732	0.094
	T2	18.53 ± 2.59	19.38 ± 2.87	−0.855	0.399
	**PANSS—general**				
	T0	58.27 ± 8.44	60.06 ± 11.04	−0.506	0.617
	T1	53.93 ± 8.75	54.38 ± 10.75	−0.125	0.901
	T2	49.60 ± 10.53	50.81 ± 10.06	−0.328	0.745
	**PANSS—total**				
	T0	104.67 ± 8.45	111.44 ± 13.21	−1.687	0.102
	T1	96.07 ± 9.00	102.50 ± 12.42	−1.642	0.111
	T2	88.27 ± 11.57	95.94 ± 11.06	−1.887	0.069
	**GAF**				
	T0	50.67 ± 4.61	46.69 ± 4.44	2.449	0.021
	T1	54.40 ± 4.21	51.06 ± 3.89	2.296	0.029
	T2	59.80 ± 3.57	57.50 ± 4.38	1.596	0.121
	**DES**				
	T0	25.53 ± 2.20	33.50 ± 6.00	−4.967	0.000
	T1	21.07 ± 3.08	31.00 ± 5.29	−6.330	0.000
	T2	19.73 ± 3.04	30.25 ± 5.09	−6.924	0.000
**DUP ≥ 1 year**		**CUD− (*n* = 15)**	**CUD+ (*n* = 16)**	***t***	***p***
	**PANSS—positive**				
	T0	29.87 ± 5.30	32.38 ± 5.35	−1.310	0.201
	T1	27.47 ± 4.26	30.63 ± 5.14	−1.857	0.074
	T2	26.27 ± 5.08	29.94 ± 5.27	−1.972	0.058
	**PANSS—negative**				
	T0	28.93 ± 3.65	24.69 ± 4.19	2.998	0.006
	T1	27.07 ± 4.08	23.13 ± 3.65	2.839	0.008
	T2	26.07 ± 3.77	23.06 ± 3.89	2.181	0.037
	**PANSS—general**				
	T0	58.07 ± 6.24	58.13 ± 5.76	−0.027	0.979
	T1	53.47 ± 5.74	57.75 ± 7.45	−1.784	0.085
	T2	51.93 ± 5.76	55.50 ± 6.52	−1.609	0.118
	**PANSS—total**				
	T0	116.87 ± 8.92	115.19 ± 8.00	0.552	0.585
	T1	108.00 ± 8.15	111.50 ± 11.02	−1.000	0.326
	T2	104.27 ± 7.60	108.50 ± 9.84	−1.334	0.193
	**GAF**				
	T0	49.33 ± 5.19	47.38 ± 6.46	0.926	0.362
	T1	52.33 ± 4.91	48.69 ± 5.20	2.004	0.054
	T2	56.27 ± 3.24	53.19 ± 2.61	2.922	0.007
	**DES**				
	T0	29.80 ± 5.19	38.50 ± 7.08	−3.881	0.001
	T1	28.53 ± 5.21	37.31 ± 7.56	−3.740	0.001
	T2	24.73 ± 4.76	35.25 ± 8.35	−4.270	0.000

Statistics: Student’s *t*-test; all results are reported as mean ± SD.

## Data Availability

The data presented in this study are available on request from the corresponding author. The data are not publicly available due to privacy reason.
